# In Vivo Metabolic Regulation of Alternative Oxidase under Nutrient Deficiency—Interaction with Arbuscular Mycorrhizal Fungi and *Rhizobium* Bacteria

**DOI:** 10.3390/ijms21124201

**Published:** 2020-06-12

**Authors:** José Ortíz, Carolina Sanhueza, Antònia Romero-Munar, Javier Hidalgo-Castellanos, Catalina Castro, Luisa Bascuñán-Godoy, Teodoro Coba de la Peña, Miguel López-Gómez, Igor Florez-Sarasa, Néstor Fernández Del-Saz

**Affiliations:** 1 Laboratorio de Fisiología Vegetal, Departamento de Botánica, Facultad de Ciencias Naturales y Oceanográficas, Universidad de Concepción, 4030000 Concepción, Chile; jose.m.ortiz.g@gmail.com (J.O.); csanhuez@gmail.com (C.S.); catalinacastro@udec.cl (C.C.); lubascun@gmail.com (L.B.-G.); 2 Centro de Estudios Avanzados en Fruticultura (CEAF), Camino Las Parcelas 882, km 105 Ruta 5 Sur. Sector los Choapinos, 2940000 Rengo, Chile; a.romeromunar@gmail.com; 3 Department of Plant Physiology, Faculty of sciences, University of Granada, 18071 Granada, Spain; javierhidalgo@ugr.es (J.H.-C.); mlgomez@ugr.es (M.L.-G.); 4 Centro de Estudios Avanzados en Zonas Áridas (CEAZA), 1700000 La Serena, Chile; teodoro.cobadelapena@ceaza.cl; 5 Centre for Research in Agricultural Genomics (CRAG) CSIC-IRTA-UAB-UB, Campus UAB Bellaterra, 08193 Barcelona, Spain; igor.florez@cragenomica.es

**Keywords:** alternative oxidase, arbuscular mycorrhizal fungi, nitrogen and phosphorus nutrition, rhizobium, plant primary metabolism

## Abstract

The interaction of the alternative oxidase (AOX) pathway with nutrient metabolism is important for understanding how respiration modulates ATP synthesis and carbon economy in plants under nutrient deficiency. Although AOX activity reduces the energy yield of respiration, this enzymatic activity is upregulated under stress conditions to maintain the functioning of primary metabolism. The in vivo metabolic regulation of AOX activity by phosphorus (P) and nitrogen (N) and during plant symbioses with Arbuscular mycorrhizal fungi (AMF) and *Rhizobium* bacteria is still not fully understood. We highlight several findings and open questions concerning the in vivo regulation of AOX activity and its impact on plant metabolism during P deficiency and symbiosis with AMF. We also highlight the need for the identification of which metabolic regulatory factors of AOX activity are related to N availability and nitrogen-fixing legume-rhizobia symbiosis in order to improve our understanding of N assimilation and biological nitrogen fixation.

## 1. Introduction

Nitrogen (N) and phosphorus (P) are the two essential macronutrients for plants. Short supply of these nutrients may lead to the appearance of stress symptoms affecting photosynthesis, respiration and, thus, plant growth [1,2,3,4,5,6]. Under abiotic stress conditions, oxygen consumption in mitochondria may be less constrained than carbon fixation in chloroplasts due to the nature of the non-phosphorylating alternative pathway of respiration. This can help to maintain the functioning of primary metabolism and carbon balance even when photosynthesis is severely restricted [7,8]. This singularity of the respiratory behavior can be especially notorious in roots under nutrient deficiency because respiration may increase to satisfy high carbon costs of nutrient uptake [8,9,10]. Roots are a sink for carbohydrates due to the energy requirements for ion transport, nutrient assimilation, growth and maintenance [3,11,12,13]. Although alternative respiration is linked to carbon respiratory losses detrimental for plant growth, roots subjected to nutrient deficiency can reduce the energy efficiency of respiration through a respiratory bypass via alternative oxidase (AOX) as part of a coordinated response directed to maximize the efficiency of nutrient acquisition. Mycorrhizas and N_2_-fixing legume root nodules are recognized as the two major plant root symbioses for enhancing nutrient uptake and plant nutrient status [14,15]. The regulation of AOX activity in plants during symbiosis is of vital importance for the determination of both the energy efficiency of respiration and the costs of carbon and energy (ATP) associated with plant symbioses. This knowledge can be of great interest for breeding programs to improve crop production through plant symbiosis with soil microorganisms. Although this line of research is still in its infancy, recent studies have evaluated the metabolic regulation of the in vivo AOX activity in leaves and roots of plants in symbiosis with soil microorganisms, as will be discussed in the present review. Their observations have provided first evidences of how alternative respiration of both plant organs can be affected in the presence of microbial symbionts.

Plant respiration is the combination of redox reactions, mostly involving both the mitochondrial tricarboxylic acid (TCA) cycle and electron transport chain (mETC), which produce carbon skeletons, carbon dioxide (CO_2_) and ATP coupled to the consumption of oxygen (O_2_) and reducing equivalents [NAD(P)H and FADH_2_] through the activities of two respiratory pathways that compete for electrons from the ubiquinone (UQ) pool [16]. The cytochrome oxidase pathway (COP) is the primary respiratory pathway, while the alternative oxidase pathway (AOP) decreases the energy efficiency of respiration. AOX contributes to dissipate the excess of reducing equivalents from chloroplasts and mitochondria and provides metabolic flexibility when COX is impaired under several abiotic stressors [8]. The in vivo electron partitioning between the two pathways and the activities of cytochrome oxidase (COX) and AOX can be measured by using the oxygen-isotope fractionation technique that allows measurements of O_2_ consumption combined with its isotopic modification during plant respiration [17]. Over the last couple of decades, the regulation of AOX activity under a large range of abiotic and biotic stresses has been extensively studied in plants as recently reviewed by Del-Saz et al. [8]. In the last few years, several studies have focused on the in vivo AOX regulation at the post-translational level, reporting simultaneous changes in both AOX activity and levels of metabolites belonging to different metabolic pathways that produce and/or dissipate reducing equivalents [8]. These observations suggest that AOX activity can confer the metabolic flexibility needed for the continuity of primary metabolism, protein turnover and plant growth under stress [8,18].

Plant symbioses with soil microorganisms may increase plant growth and affect levels of primary metabolites through the exchange of carbon and nutrients between plant roots and microsymbionts [19,20]. The enhancement of plant growth is due to a double effect. On the one hand, the microsymbiont induces an increase of nutrient content in plant organs, increasing carbon assimilation during photosynthesis [21,22,23]. On the other hand, increased rates of photosynthesis allow plants to satisfy the microsymbiont’s demand for carbon compounds. In other words, rates of photosynthesis are incremented (due to the decrease of photosynthetic limitations) because microsymbiont leads plants to produce large amounts of carbon compounds required for its metabolism [19]. This phenomenon, called positive-feedback of photosynthesis [24], could be accompanied by adjustments in the energy efficiency of respiration, considering the tight relationship between photosynthesis and AOX activity [25,26,27,28]. Thus, adjustments in the energy efficiency of respiration can be conditioned by the metabolic costs of microsymbiont maintenance or by the metabolic benefits of improved nutrition, which in turn may depend on the microbial symbiont’s energy efficiency of respiration.

## 2. Regulation of AOX Activity by P Availability

The main P resource for plants in soils is inorganic phosphate (Pi), which mostly can be retained or complexed by cations (e.g., Ca^2+^ and Mg^2+^) [29]. The other P pool in soil comprises organic P compounds derived from the degradation of plant litter, microbial detritus and organic matter [30]. Pi is involved in cellular bioenergetics and metabolic regulation, and it is also important as a structural component of essential biomolecules such as DNA, RNA, phospholipids, ATP and sugar-phosphates [2,31]. A decrease in cytosolic Pi may restrict oxidative phosphorylation, leading to an increased proton gradient and membrane potential. In turn, this prompts an over-reduction of the components of the electron transport chain, inhibiting oxygen consumption through the COX pathway, which is coupled with ATP synthesis. This creates a decrease in the re-oxidation of NADH produced in the TCA cycle [4,16]. Furthermore, the accumulation of NADH in the mitochondrial matrix also inhibits the TCA cycle dehydrogenases, decreasing the activity of the TCA cycle and limiting the production of important metabolic intermediates [32].

A plant trait that enhances the capacity to acquire P in the poorest P soils is the production of cluster roots in members of the Proteaceae family, most of which do not form mycorrhizal associations [33,34,35,36]. Cluster roots are very effective at acquiring P that is largely absorbed into soil particles, because of their pronounced capacity to exude carboxylates [9]. Cluster roots of *Lupinus albus* release much more citric and malic acid than lupin roots of plants grown under P sufficiency. Florez-Sarasa et al. [37] observed that growth under P limitation increased the activity of AOX in cluster roots of *L. albus* together with the synthesis of citrate and malate. This is in the line with previous studies describing an incremented AOX abundance in cluster roots of *Hakea prostata* [38] and in suspension cells of tobacco after exogenous supply of citrate [39]. It is thought that the production of vast amounts of citrate in cluster rootlets is inexorably associated with the production of NADH [37,38,39,40]. This led Florez-Sarasa et al. [37] to state that AOX allows the continuity of TCA cycle activity by re-oxidizing the high levels of NADH produced during citrate synthesis when COX activity is restricted due to the P-deficiency-induced adenylate restriction.

**Figure 1:**
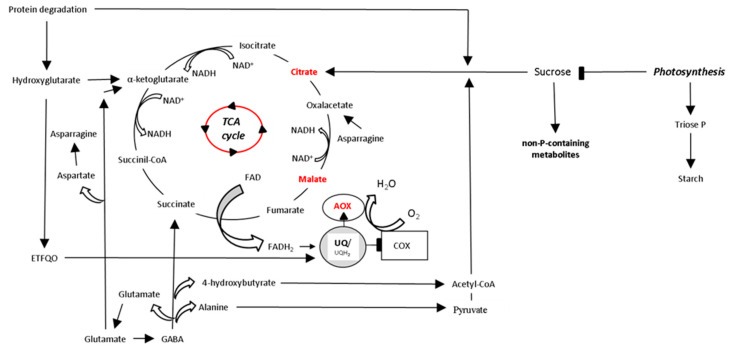
Schematic representation of the TCA cycle and its connection with Pi consumption in leaves of plant species adapted to P deficiency. Low Pi availability limits both photosynthesis and respiration. In chloroplasts, the export rate of the Calvin-Benson cycle products, which are needed for the synthesis of sucrose, decreases under P limitation. This leads to increasing amounts of triose-phosphate and starch in chloroplasts. In cytosol, an accumulation of non-P-containing saccharides allows the cell to save Pi, but it aggravates the short supply of respiratory substrates into mitochondria. In contrast, protein degradation provides carbon skeletons to mitochondria via hydroxyglutarate synthesis that can be used for the synthesis and exudation of rhizosphere carboxylates citrate and malate, and feeds electrons to the mETC through to the ubiquinol pool via an electron-transfer flavoprotein:ubiquinone oxidoreductase (ETFQO) [56]. Similarly, the γ-aminobutyrate (GABA) shunt allows the entry of carbon skeletons in the form of acetyl-CoA, pyruvate, succinate, oxalacetate and α-ketoglutarate into the TCA cycle from amino acids alanine, glutamate and asparagine [58]. The re-oxidation of NADH generated in the TCA cycle may be favored by AOX activity when COX is restricted by low Pi availability. TCA, tricarboxylic acid cycle.

The capacity to synthesize acidifying and/or chelating compounds is not restricted to species with morphological structures such as cluster roots and dauciform roots, although it is less abundant [9]. In roots without these adaptations, and in the absence of mycorrhiza, the levels of enzymes involved in organic acid biosynthesis, such as PEP carboxylase, often increase in response to P starvation in pea, tomato and *Brassica nigra* [41]. This increase in enzyme levels was related to a higher amount of organic acids being produced for root exudation. This capacity is not only present in roots; leaves of plants grown under P limitation may accumulate carboxylates such as citrate, malate and fumarate [41,42]. Carboxylates in leaves can be transported via the phloem and directed to roots for exudation [41,42]. Pioneering studies reported an adaptive response of respiratory metabolism and the mitochondrial electron transport chain to P limitation in NM roots [43,44,45,46], including increased AOX capacity [44,46,47,48,49]. This in the line with previous studies reporting imbalances of C/N ratio and ROS levels in AOX-deficient cells under P deficiency [46,48], although the situation at tissue level has been recognized to be more complex [18]. Recent studies have observed increases of AOX activity in roots of non-cluster roots for species grown under P limitation, such as *Nicotiana tabacum* and *Solanum lycopersicum* in the absence of mycorrhiza [13,50]. In these species, increments of AOX activity were observed, coinciding with a higher synthesis of carboxylates citrate and malate. In leaves, there were reports of pioneer studies reported increases of AOX activity in *Phaseolus vulgaris* and *Gliricidia sepium* plants grown under P limitation, but a decrease of foliar AOX activity was observed in *Nicotiana tabacum*, although this disparity was not related to any respiratory metabolite [51]. A recent study in *Solanum lycopersicum* plants grown at P-sufficient and limiting conditions, and exposed to sudden short-term (24 h) P-sufficient pulse, observed foliar respiratory bypasses via AOX and an increased accumulation of citrate, together with an enhanced expression of high-affinity P transporters *LePT1* and *LePT2* in conditions of limited P concentration [50]. These observations suggest that P concentration in plant organs regulates AOX activity in coordination with biochemical and molecular adjustments, functioning as a mechanism directed to maximize P acquisition [50]. Despite these findings, there is still a lack of understanding about the entire metabolic puzzle leading to the synthesis of citrate and increases in AOX activity. Studies combining metabolite profiling and measurements of electron partitioning between COX and AOX in P deficient plants could certainly shed light on the metabolic role of AOX in plant species adapted to P deficiency, which increase carbon use efficiency by decreasing Pi consumption in leaves as represented in Figure 1, below. The rate of photosynthesis and the export of its products from the chloroplast are determined by the availability of Pi in both chloroplast and cytosol [9,52]. Low chloroplast Pi availability induces a decrease in the rate of photosynthesis by decreasing both ATP synthesis and Calvin-Benson cycle activity, which results in a reduced availability of intermediates, e.g., ribulose 1,5-biphosphate (RuBP), and decreased carboxylation activity of Rubisco [53]. Low cytosolic Pi availability decreases the export rate of the products of the Calvin-Benson cycle, leading to increasing amounts of triose-phosphate and starch in the chloroplast [52,53]. Consequently, sucrose formation and glycolysis can be reduced, which may limit carbon supply into mitochondria, thus decreasing both TCA cycle activity and respiration [5,30], and therefore, plant growth and yield. In order to save Pi, leaves reduce Pi consumption in phosphorylation of sugar metabolites by converting phosphorylated metabolites (glucose-6-P, fructose-6-P, inositol-1-P and glycerol-3-P) to non-P-containing di- and tri-saccharides, as observed in *Hordeum vulgare* and *Eucalyptus globulus* P-deficient plants [54,55]. In these studies, such changes coincided with reduced levels of organic acid intermediates of the TCA cycle, suggesting a short entry of carbon into mitochondria. Bearing in mind that the conversion of di- and tri-saccharides to organic acids requires Pi, it is unlikely that they can be further respired [54]. Under this circumstance, the use of alternative carbon resources would allow the continuity of TCA cycle reactions to produce organic acids, e.g., citrate for secretion and to sustain the mitochondrial electron transport chain. In this sense, changes in levels of amino acids glutamine, arginine and asparagine was observed in P-deficient plants [54,55]. A similar response was recently observed in *Hordeum vulgare* [42]. These amino acids were suggested to provide carbon skeletons to mitochondria when plants reduce the consumption of Pi [42]. It is known that plants can metabolize proteins and lipids as alternative respiratory substrates when carbohydrates are scarce in plant cells [56,57,58]. Carbon consumption of these alternative respiratory substrates could be associated with the generation of NADH in the TCA cycle, whose re-oxidation would be favored by AOX activity when COX is restricted under P deficiency (Figure 1).

### Regulation of AOX Activity by Arbuscular Mycorrhizal Symbiosis

More than 90% of terrestrial plants are associated with root-colonizing fungi, establishing a durable and close mutualistic symbiosis, called mycorrhiza [59]. The endotrophic arbuscular mycorrhiza is the most common type, occurring in about 80% of plant species [60]. The establishment of the association between AMF and plants implies the generation of roots with representative structures typical of this symbiosis such as (1) intraradical mycelium, which is a fungal structure that inhabits the plant intracellular space; (2) arbuscule, which is the space where the carbon and nutrient exchange between fungus and plant takes place; (3) the vesicles, storage structures; and (4) the extraradical mycelium, which is a structure that extends from the root surface to the soil, beyond the root P-depletion zone and has access to a greater volume of soil compared to roots and root hairs alone [61]. Mycorrhizal associations act as ‘scavengers’ for Pi uptake in the soil solution. Compared to non-mycorrhizal (NM) plants, the advantages of increased P acquisition and photosynthesis increase with decreasing soil P availability [62]. The increase in photosynthesis in plants with mycorrhiza is related to an increased demand for carbohydrates supplied to the fungus [19,63]. Some carbohydrates produced in leaves during photosynthesis are transported to roots, where they are broken down in respiration to produce ATP and carbon skeletons required for protein synthesis. Around 20% of the carbon fixed by photosynthesis is destined to form soluble sugars and organic acids in order to supply energy metabolism in fungal cells [64]. These metabolic carbon requirements of AM symbiosis may affect plant respiration [65,66,67,68] as well as the levels of primary metabolites in plant organs [69,70,71]. In fact, AM symbiosis decreases the carboxylate-releasing strategy as observed in 10 *Kennedia* species and five species of legumes [72,73]. The mechanism for the reduction in rhizosphere carboxylates with AM symbiosis could be a consequence of the reduction of carbon availability in roots due to the demand of AMF for carbon compounds, or it could be a consequence of higher plant P concentration due to improved nutrition. Measurements of in vivo AOX activity and the accumulation of carboxylates in roots of *Nicotiana tabacum* and *Arundo donax,* showed that AM symbiosis decreased root respiration via COX and AOX in *N. tabacum*, decreased respiration via COX in *A. donax*, and decreased synthesis and exudation of citrate and malate in *A. donax* and *N. tabacum*, respectively [13,74]. On top of this, both species showed symptoms of ameliorated physiological status and increased biomass accumulation in shoots. These results probably denote that the synthesis of rhizosphere exudates in non-AM plants imposes an important carbon cost detrimental for plant growth as compared with AM plants, which do not invest as much carbon in the synthesis of carboxylates, thus respiring less and allowing carbon to accumulate. Bearing all this in mind, it would be logical to assume that the mechanism for the reduction in rhizosphere carboxylates is related to improved plant P status rather than less carbon availability. In fact, previous studies described that increasing P availability tends to reduce the amount of carboxylate in rhizosphere soil [75,76], and the carboxylate-releasing strategy requires more carbon when P availability is in the range at which AM plants are functional [77]. Nevertheless, it is important to highlight that the effect of AM symbiosis on plant growth is variable because it depends on the host plant and the fungal species [78]. In this sense, in vivo AOX measurements have been made only in positive symbiotic interactions (beneficial for plant growth), and there are still a lack of studies that test the role of alternative respiration in defective symbiotic interactions (detrimental for plant growth). Moreover, it has been reported that the effect of AMF on plant growth depends on the stage of colonization [61]. In this sense, a recent study in *N. tabacum* showed that symbiosis with *Rhizophagus irregularis* differently affects both respiration and ATP synthesis in leaves at different growth stages when plants grow in P deficient soils. AM symbiosis represented an ATP cost (via decreased COX activity) for tobacco leaves that was detrimental for shoot growth at early stages, presumably because fungal structures were still under construction. At the mature stage, this cost turned into an ATP benefit (via incremented COX activity), which allowed for faster growth presumably because symbiosis was functional, bearing in mind the observed increase in both foliar P status and shoot growth [79].

**Figure 2:**
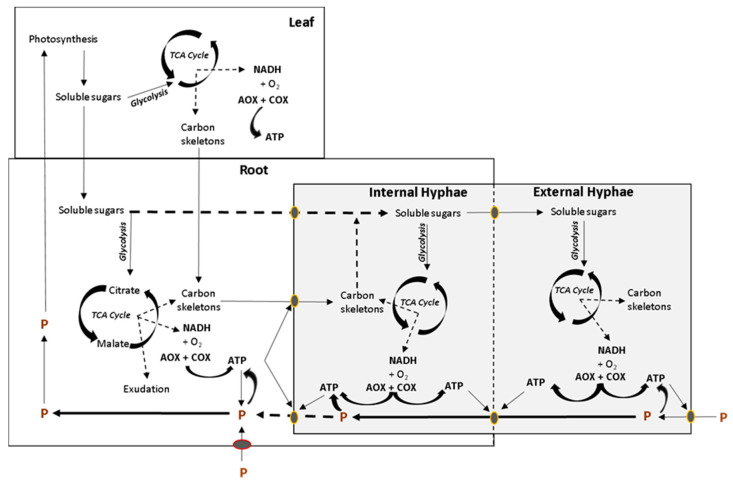
Simplified overview of the interaction between respiratory metabolism of plant organs and mycorrhiza, conditioned by the demand for ATP synthesis and P uptake. Photosynthetic soluble sugars are used in respiration in leaves or transported to the root in order to fuel respiration and produce carbon skeletons for the fungal symbiont. Soluble sugars and organic acids can be exported to the fungal symbiont to fuel respiration in both intra and extraradical mycelium. ATP is required for P uptake and transport across organisms. TCA, tricarboxylic acid cycle. Modified from Hughes et al. [60].

AM symbiosis can improve nutrient acquisition because AM provide an additional means of nutrient uptake, the mycorrhizal nutrient uptake pathway [80,81], which can bypass the pathway of direct nutrient uptake in a P availability-dependent manner [82,83,84,85,86,87]. Studies relating the functioning of the mycorrhizal nutrient uptake pathway to the in vivo electron partitioning to AOX are required, keeping in mind that AOX is also present in various fungi including *Rhizophagus intraradices* [88], and that P acquisition by AMF requires energy, which is obtained during oxidative phosphorylation in fungal mitochondria. Precisely, ATP is needed for P uptake by the external hyphae, P transport and export to the internal hyphae and P uptake by the plant at the arbuscule (Figure 2). It would be logical to assume that positive AMF-plant interactions display high rates of COX activity in extra radical mycelium to ensure ATP availability and to energize the mycorrhiza pathway uptake. Measurements of the in vivo COX and AOX activities together with techniques such as multicompartment plant growth systems [89] and ^13^C and ^33^P isotopic labeling [90] may help to identify AMF-plant associations with efficient energy rates of extra radical mycelium respiration when the mycorrhizal nutrient uptake pathway is active. This could contribute to expand our view on the interplay between nutrient uptake pathways in plants with mycorrhiza.

## 3. Regulation of AOX Activity by N Availability

Nitrogen is a major component of the photosynthetic apparatus and is required by plants in greater quantities than any other mineral element. Almost all the N available for plants is present in the reduced form of nitrate (NO_3_^−^), ammonium (NH_4_^+^), organic compounds and molecular nitrogen (N_2_) in the air [91,92]. The major source of N in soils resides in the atmosphere, through both biological N_2_ fixation and the deposition of NO_3_^−^ and NH_4_^+^ in precipitation. In soils, NH_4_^+^ and NO_3_^−^ move towards roots through transpiration-driven mass flow because they are water soluble [93]. Both NH_4_^+^ and NO_3_^−^ enter the plant cells via specific transporters [94,95]. In order to be incorporated, NO_3_^−^ is reduced to NH_4_^+^ by nitrate reductase (NR) and nitrite reductase [96]. Then, NH_4_^+^ is further converted into glutamine and glutamate, in a reaction catalyzed by glutamine synthetase/glutamine 2-oxoglutarate amino-transferase (GS/GOGAT) cycle [95]. At the cellular level, nitrogen assimilation is finely regulated according to its supply and demand. Nitrogen controls the regulation of nitrate transporters, activities of nitrate and nitrite reductase, the functioning of primary metabolic pathways associated with the production of reducing equivalents and the production of organic acids required for N assimilation into amino acids [2].

There is a correlation between leaf N content and rates of respiration [97,98,99,100]. Short supply of N leads to decreasing rates of both photosynthesis and respiration by reducing protein turnover and increasing breakdown of nucleic acids and enzymes [9,101]. Under these circumstances, a minimum supply of TCA metabolites (e.g., 2-oxoglutarate, isocitrate, and citrate) may maintain optimum N assimilation and amino acid biosynthesis [102,103]. Indeed, TCA cycle enzymes fumarase, NAD-dependent isocitrate dehydrogenase, and NAD-dependent malic enzyme are up-regulated under low N [98,104], and protein degradation acts as an alternative respiratory substrate [56]. In this situation, AOX activity could play a role in maintaining the functioning of primary metabolism by allowing adjustments in energy efficiency of respiration. However, there are no studies that test the regulation of AOX activity in vivo under N limitation. Such a hypothetical role should be evaluated in plant species with constitutively high levels of both AOX protein and activity. Plants possess an AOX overcapacity [8,26,105,106,107], which is variable depending on the plant species and environmental conditions [26,107]. It is thought that this overcapacity eliminates the need for de novo AOX protein synthesis, granting alternative respiration the ability to respond to sudden changes in levels of reducing equivalents [8,26,107]. In this sense, a short supply of N in plant species with high levels of AOX protein, such as legumes, could induce a decrease of both AOX protein and capacity to some extent without compromising the accuracy of AOX activity measurements, allowing us to evaluate the in vivo role of AOX under stress. Preliminary results from our laboratory in *Lotus japonicus* have shown that total respiration decreases (via COX and AOX) in leaves and roots when plants grow under short supply of KNO_3_, in comparison with plants grown at sufficient KNO_3_ supply. Interestingly, a short supply of KNO_3_ induces an increase of the energy efficiency of respiration (via decreased contribution of AOX activity to total respiration) only in leaves (unpublished), which suggests the existence of a differential regulation between organs directed to maximize ATP synthesis in leaves, most likely for maintenance purposes.

**Figure 3:**
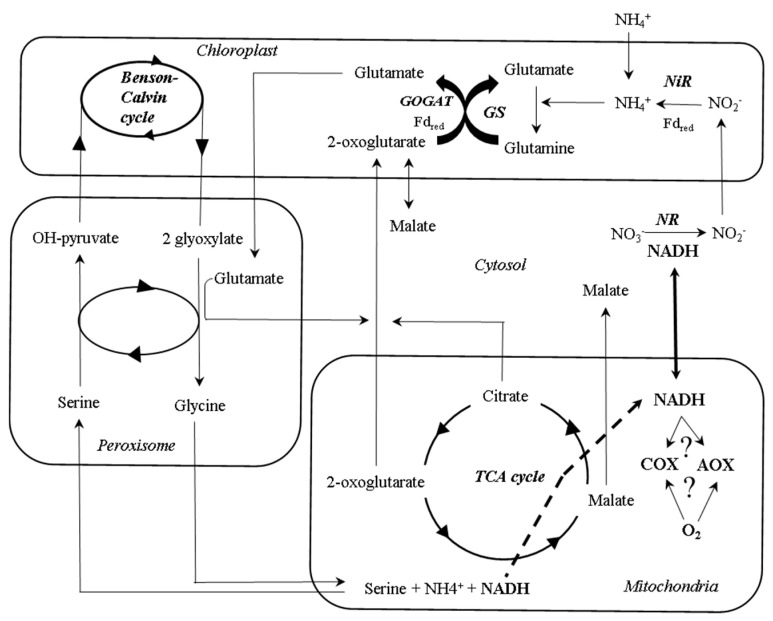
A simplified schematic overview of the compartmentation of some of the interactions between primary metabolism pathways during ammonium and nitrate assimilation. Nitrate is mainly transported from roots to leaves via xylem, where it is converted into nitrite with the consumption of reducing equivalents in cytosol. In the chloroplast, the reducing power of light-activated electrons drives the conversion of nitrite to ammonium from cytosolic nitrate reductase (NR)-derived nitrite by a nitrite reductase (NiR) activity, and its assimilation by the GS/GOGAT cycle. 2-oxoglutarate which is required for ammonium assimilation, is exported to the chloroplast by a 2-oxoglutarate/malate translocator. Ammonium uptake bypasses the nitrate reductase reaction in cytosol, thus increasing the reducing equivalents available that can be dissipated during respiration. During photorespiration, the retrieval of CO_2_ and NH_4_^+^ during the glycine cleavage reaction in mitochondria leads to an increased NADH/NAD^+^ ratio in the mitochondrial matrix that has been suggested to be related to changes in AOX activity. TCA, tricarboxylic acid.

On the other hand, the source of N could be another regulatory factor of AOX activity in leaves. Classic studies have observed that the expression and activity of several glycolytic and TCA cycle enzymes were differentially affected following NH_4_^+^ or NO_3_^−^ uptake [102,103,108], which could be accompanied with respiratory adjustments. It is known that nitrate uptake and its conversion to ammonium require large amounts of ATP and reducing equivalents [109,110,111]. However, respiration increases when ammonium is present as the main N source [112,113,114]. This has been explained as a consequence for the lack of the important reductant sink exerted by nitrate reductase, thus leading to an increase of reducing equivalents in cytosol, that are dissipated by mitochondrial electron transport chain. Under this scenario, AOX could play a significant role during this dissipation of reductants, considering its roles in maintaining the cell redox balance [8,115,116]. In this way, the accumulation of NH_4_^+^ and its associated toxicity is prevented by the action of the GS/GOGAT cycle activity [117] in parallel to the mitochondrial dissipation of reductants. In fact, previous studies have shown an increase in AOX capacity and enhanced of several AOX isoforms in plants grown under NH_4_^+^ supply [114,115,118,119,120]. Moreover, negative correlations between AOX capacity and nitrate concentrations were observed [114], although the electron flow through AOX under aerobic conditions can be important for the reduction of NO generation associated to nitrate reduction [121]. Interestingly, the growth of AOX-overexpressing plants is less restricted as compared to wild type (WT) *Arabidopsis* plants grown under NH_4_^+^ nutrition, although the metabolic causes of this phenotype remain uncertain [118]. The hypothetical role of AOX in conferring metabolic flexibility during NH_4_^+^ nutrition still needs to be tested by in vivo activity measurements (Figure 3).

### Regulation of AOX Activity in the Rhizobium-Legume Symbiosis

Legumes are good candidates to study the regulation of AOX activity by N availability because these plants have been suggested to display faster rates of foliar AOX activity under stress as they constitutively express high levels of AOX protein under normal growth conditions [122,123,124]. In fact, measurements of in vivo AOX activity have been performed in leaves of six legumes species: common bean (*Phaseolus vulgaris*), garden pea (*Pisum sativum*), barrel medic (*Medicago truncatula*), soybean (*Glycine max*), mung bean (*Vigna radiata*) and faba bean (*Vicia faba*). These experiments have been important to evaluate the regulation of the AOX activity under P limitation, high light, salinity, pathogen infection and variable temperatures [25,26,27,51,106,125,126]. Furthermore, legumes are suitable for the study of the regulation of AOX activity by N availability in roots because of their ability to establish symbiosis with a group of soil bacteria collectively designated as rhizobia. Rhizobia is a group of diazotrophs, most of them belonging to the α-proteobacteria, that include the genera *Rhizobium*, *Mesorhizobium*, *Ensifer* (formerly *Sinorhizobium*), *Bradyrhizobium* and *Azorhizobium*, among others [127]. The rhizobia-legume symbiosis provides a suitable biological system to evaluate variations in both nutrient status and metabolite levels in plant organs due to the exchange of carbon and nutrients between host plants and bacteroids (that is, the differentiated endosymbiotic form of the bacteria able to fix nitrogen).

**Figure 4:**
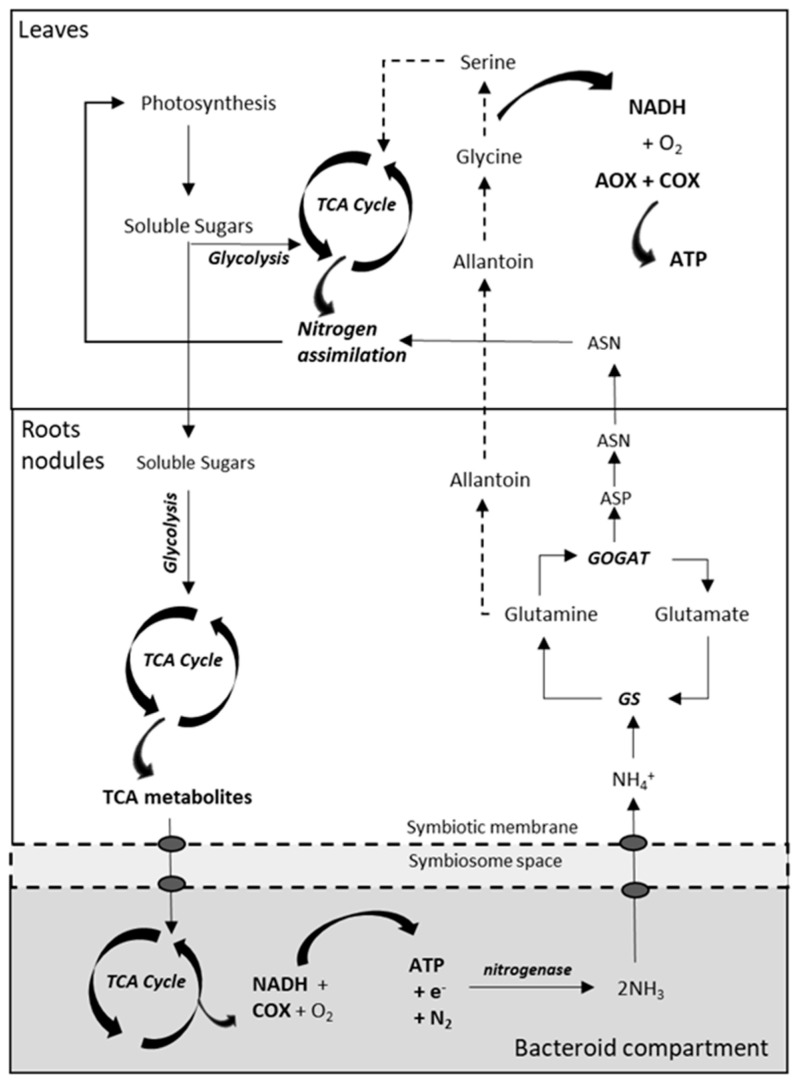
Simplified overview of the nitrogen-fixing pathways in nodulated legumes, conditioned by the demand for ATP synthesis for nitrogenase activity in determinate and indeterminate nodules. Soluble sugars are catabolized via glycolysis to respiratory substrates for the synthesis of TCA metabolites, which are transported across the peribacteroid and bacteroid membranes to fuel the TCA cycle and respiration in the bacteroid. The ammonia produced during nitrogenase activity is exported to the plant and assimilated by GS and GOGAT enzymes. In determinate nodules, glutamine is converted to ureides (allantoin), that are decarboxylated in metabolic pathways of photorespiration, contributing to the accumulation of NADH in mitochondria. In indeterminate nodules, glutamine and glutamate are further converted to asparagine and aspartate to be incorporated into the nitrogen metabolism of leaves. ASN, asparagine; ASP, aspartic acid; TCA, tricarboxylic acid cycle. Modified from Liu et al. [131].

Legumes can fix atmospheric nitrogen (N_2_) through the nitrogenase activity that reduces N_2_ to NH_3_^−^ and is located in the root nodule bacteria [128,129,130]. Biological N_2_ fixation in leguminous plants requires the development of a specific symbiotic relationship between rhizobia soil bacteria and the plant root in conditions of limited nitrogen availability in soil [131]. In bacteroids, the nitrogenase reaction requires a great deal of energy, consuming at least 16 ATP and four pairs of electrons for every molecule of N_2_ reduced to ammonia [131,132]. This energy is obtained from plant carbon compounds in the form of TCA cycle intermediates (fumarate, succinate or malate) via a dicarboxylic-acid transport system [132,133,134]. Similar to mycorrhizal symbiosis, the nodule imposes a carbon cost in roots that cannot exceed their nutritional benefit. However, it is unknown whether respiratory adjustments in nodulated roots contribute to the regulation of the carbon economy in legumes. In this sense, preliminary results from our laboratory obtained in roots of *L. japonicus* nodulated by *Mesorhizobium loti* revealed higher rates of total respiration via COX (and diminished AOX activity) when compared to non-nodulated roots of plants grown at low KNO_3_ (unpublished). These results are in agreement with the previous studies describing high rates of respiration in nodulated roots [135,136,137]. On the other hand, we observed similar rates of respiration in nodulated roots when compared to non-nodulated roots of plants grown at sufficient KNO_3_ (unpublished). Based on these results, it seems that the effect of rhizobia on root respiration could be related to an improved N status rather than to carbon costs of nodule maintenance and nitrogenase activity. Biological nitrogen fixation leads to the production of ammonium in bacteroids, which is transferred to the host plant through the symbiosome membrane and initially assimilated to glutamine, and then to either ureides or amides to ameliorate N status in leaves [130,134,138,139,140]. Similar to roots, rhizobia inoculation did not significantly change the activities of the two terminal oxidases in leaves of *L. japonicus* plants grown under KNO_3_ sufficiency, thus suggesting a similar N status between these plants. Furthermore, leaves of non-nodulated plants grown at low KNO_3_ displayed the lowest rates of ATP synthesis via decreased COX and AOX (unpublished). Based on these preliminary results, it seems that the activities of both COX and AOX in plant organs depend on N availability. Another regulatory factor of AOX activity in leaves could be determined by the type of nodule. The determinate legume root nodules, characteristic of some tropical legumes as soybean and common bean, primarily exports ureides (allantoin and allantoate) as fixed-N compounds to be metabolized in leaves. These compounds are converted to glycine, which in turn will be converted to serine as part of the photorespiration pathway that is associated to the mitochondrial release of ammonia in and its re-assimilation into nitrogenated compounds [133,141]. On the other hand, indeterminate nodules, characteristic of certain temperate legumes as barrel medic and pea, assimilate amides in the form of asparagine (Asn) and glutamine (Gln) [141,142], which are exported to the aerial part to be directly incorporated into leaf metabolism. This bypasses the production of reducing equivalents related to the decarboxylation of glycine to serine in leaf mitochondria [12] that is observed in determinate nodules, which could be associated with changes in AOX activity (Figure 4).

Although nitrogenase enzyme requires O_2_ for ATP synthesis, this enzyme is extremely O_2_-labile, being inhibited above a certain O_2_ concentration. This was called “the oxygen paradox” [143]. In order to maintain respiration and ATP synthesis in the infected cells, the nodule displays several mechanisms for delivering a regulated flux of O_2_, while maintaining a free O_2_ concentration at low levels in infected cells. One of these mechanisms is the occurrence of an O_2_ diffusion barrier to the nodule central zone, where nitrogen-fixation takes place [144]. Moreover, it is thought that rates of bacteroid respiration are high enough to ensure a quick consumption of O_2_, as soon as the gas diffuses into the central zone to avoid its accumulation [145]. However, fast rates of nitrogenase activity would increase the demand for O_2_ concentration in the infected cells. To increase O_2_ diffusion to bacteroids, the infected cells contain leghemoglobin that acts as an O_2_ carrier. This is an iron protein with high affinity for O_2_, which regulates O_2_ diffusion from the cytosol to the bacteroid in adequate concentrations to fuel its respiration, preventing inhibition of nitrogenase [146,147]. Thus, the ability of the nodule to respond to sudden increases of O_2_ in infected cells is very important because of their repercussions on biological nitrogen fixation. AOX has been found in nodules infected by several species of rhizobia such as *Bradyrhizobium japonicum* and *Rhizobium leguminosarum* [148,149], although with lower abundance and capacity than any other tissue of the same plant as was described in soybean root nodules [149]. Thus, it is unlikely that AOX activity may play a significant role in nodule respiration as it does in plant cells [149,150]. However, the observed upregulation of AOX mRNA levels in senescent bean nodules was proposed to contribute to the redox balance in mitochondria [148]. Presently, there are no results of in vivo activities of COX and AOX in legumes nodules. Accurate estimates of the activities of COX and AOX in plant nodules would require in vivo measurements in bacteroid and mitochondria by using on-line liquid-phase systems [150,151,152]. They are worthy for the corroboration of the high energy efficiency of nodule respiration, which can be assumed to be tightly coordinated with the nitrogenase activity. This is because nodule mitochondria contain COX enzyme with high affinity for oxygen and nitrogenase activity depends on O_2_ consumption for ATP synthesis [153,154,155,156,157]. In fact, abiotic stressors result in the inhibition of carbon metabolism in host legumes as well as in the increase of nodule resistance to O_2_ diffusion in order to constrain respiration and nitrogenase activity and save carbon that will eventually become scarce [158]. It is worth mentioning the existence of different metabolic responses to Pi deficiency observed among legumes when biological nitrogen fixation is suppressed under Pi deficiency [133]. One of these responses is the enhancement of Pi uptake and recycling in nodules [139,159,160] that may lead to the use of alternative respiratory substrates of carbon compounds such as amino acids, as observed in the metabolic profiles performed during symbiotic nitrogen fixation in phosphorus-stressed common bean [161]. Although methodological improvements are still needed for the study of nodule respiration, it seems that measurements of both apparent nitrogenase activity and total nitrogenase activity, in combination with O_2_ isotope fractionation and metabolite profiling in plant organs, is a good starting point to understand how biological nitrogen fixation and plant respiratory metabolism are connected in legumes.

## 4. Concluding Remarks

The study of the metabolic regulation of AOX activity in plant species that establish symbioses with soil microorganisms under nutrient deficiency is important for further understanding of plant growth responses to abiotic stress and global climate change. Previous and preliminary studies analyzing the in vivo response of AOX in roots and leaves of plants in symbioses with AMF and rhizobia under conditions of P and N limitation suggest that the absence of the symbiont imposes nutritional restrictions for ATP synthesis. Hence, plant symbioses with soil microorganisms confer energetic benefits due to improved plant nutrition. The supply of N and P from the microbial symbiont to the plant depends on the ATP availability in the microbial symbiont, which is regulated by its demand for carbon compounds. This regulation could involve high energy efficient rates of respiration for the benefit of ATP synthesis in hyphae and legume roots nodules under sudden changes in the demand for carbon compounds. An improvement in plant status delays the appearance of nutrient starvation responses that may involve changes in levels of metabolites and reducing equivalents. Moreover, it decreases the contribution of AOX activity to total respiration via increased COX activity. Thus, the in vivo metabolic regulation of AOX activity in plants seems to depend on their nutritional status. The importance of plant AOX activity for the mycrosimbiont’s colonization and functioning still needs to be tested in AOX modified plants. The identification of new regulatory factors (e.g., of AOX activity) can be taken into account in breeding programs for improvement of crop production.

## Abbreviations

AMF	Arbuscular mycorrhizal fungi
AOP	Alternative oxidase pathway
AOX	Alternative oxidase
ATP	Adenosine tri-phosphate
COP	Cytochrome oxidase pathway
GOGAT	Glutamine oxoglutarate aminotransferase
GS/GOGAT	Glutamine Synthetase-Glutamate Synthase
NM	Non-mycorrhizal
NR	Nitrate reductase
mETC	Mitochondrial electron transport chain
LePT	*Lycopersicon esculentum* phosphate transporter
RuBP	Ribulose 1,5-biphosphate
TCA	Tricarboxylic acid
UQ pool	Ubiquinine pool
